# Overcoming challenges in human saliva gene expression measurements

**DOI:** 10.1038/s41598-020-67825-6

**Published:** 2020-07-07

**Authors:** Patrick Ostheim, Ales Tichý, Igor Sirak, Marie Davidkova, Marketa Markova Stastna, Gabriela Kultova, Tatjana Paunesku, Gayle Woloschak, Matthaeus Majewski, Matthias Port, Michael Abend

**Affiliations:** 1 Bundeswehr Institute of Radiobiology affiliated to the University of Ulm, Neuherbergstr. 11, 80937 Munich, Germany; 2 Department of Radiobiology, Faculty of Military Health Sciences in Hradec Kralove, University of Defence in Brno, Brno, Czech Republic; 3 Department of Oncology and Radiotherapy, University Hospital and Faculty of Medicine, Hradec Kralove, Czech Republic; 4 Department of Radiation Dosimetry, Nuclear Physics Institute of the Czech Academy of Sciences, Prague, Czech Republic; 5 Institute for Hematology and Blood Transfusion, Hospital Na Bulovce, Prague, Czech Republic; 6 Department of Radiation Oncology, Northwestern University, Chicago, IL 60611 USA; 7 Department of Urology, Bundeswehrkrankenhaus Ulm, Ulm, Germany

**Keywords:** Transcriptomics, Molecular biology

## Abstract

Saliva, as a non-invasive and easily accessible biofluid, has been shown to contain RNA biomarkers for prediction and diagnosis of several diseases. However, systematic analysis done by our group identified two problematic issues not coherently described before: (1) most of the isolated RNA originates from the oral microbiome and (2) the amount of isolated human RNA is comparatively low. The degree of bacterial contamination showed ratios up to 1:900,000, so that only about one out of 900,000 RNA copies was of human origin, but the RNA quality (average RIN 6.7 + /− 0.8) allowed for qRT-PCR. Using 12 saliva samples from healthy donors, we modified the methodology to (1) select only human RNA during cDNA synthesis by aiming at the poly(A)+-tail and (2) introduced a pre-amplification of human RNA before qRT-PCR. Further, the manufacturer’s criteria for successful pre-amplification (Ct values ≤ 35 for unamplified cDNA) had to be replaced by (3) proofing linear pre-amplification for each gene, thus, increasing the number of evaluable samples up to 70.6%. When considering theses three modifications unbiased gene expression analysis on human salivary RNA can be performed.

## Introduction

Human saliva is mainly composed of fluid and compounds produced by the major salivary glands, including the parotid, submandibular and sublingual glands, as well as the minor salivary glands. The salivary glands secrete fluid transported from serum as well as tissues surrounding the glands. Other human saliva constituents are from the oral mucosa, periodontium, and the oral microbiome^1^.

During the last two decades, saliva has become of increased interest as an easily accessible and non-invasive source of human biomarkers. Besides DNA, proteins and various metabolites, RNA has also been shown as a promising marker in other tissues and body fluids, providing complex gene expression information. Because saliva is derived from several tissue sources and that it also contains large amounts of total RNA make it one of the most attractive diagnostic, prognostic, and monitoring tools for both systemic and oral diseases^2–4^. Hereby, saliva has been shown to contain RNA biomarkers (mRNA and small RNA species) for prediction and diagnosis of several diseases^5^, especially of the oral cavity such as oral cancer^6,7^ or disorders of the salivary glands^8,9^.

Saliva aggregates information from several bodily sources. Because saliva is a plasma ultrafiltrate, this means most compounds found in blood are also in saliva, leading to the aphorism that saliva is a “mirror of the body”^10,11^. Like peripheral blood, saliva contains a broad range of hormones, enzymes, antibodies and genetic materials which have been infiltrated from blood through various transport mechanisms such as ultrafiltration, diffusion, paracellular routes and active transport^12^. It’s a mirror of systemic processes that reflect the levels of endogenous and exogenous substances like drugs. This versatility allows for the application of saliva in the diagnosis not only for salivary gland disorders and oral diseases but also for systemic conditions (e.g. pancreatic biomarkers^13^).

While the majority of cellular nucleic acids (DNA and RNA) contained in human saliva originate from the buccal mucosa, cell-free nucleic acids originate from a wide variety of sources within the body, mainly encased in exosomes^14,15^. For analysing nucleic acids originating from systemic processes, salivary supernatant would be preferable compared to whole saliva as described in some studies^16^. But isolation of exosomes is laborious, resulting in low RNA yields. As a more robust but—due to the high background—less sensitive alternative, we examined whether whole saliva could be used.

Compared to blood, whole saliva has numerous advantages: non-invasive and simple sample collection (possibly by the study subject or an untrained person), easy and repeated sampling in older people and children^2^ and simplified logistics of sample collection (e.g. storage at room temperature for many saliva sample types without concerns of clotting as for blood). These features underscore the utility of salivary biosamples as economical and fast tools for diagnostic screening and treatment monitoring (e.g., decreasing level of tumor-specific miRNAs throughout the course of cancer treatment) in routine clinical practice. Saliva sampling could be an efficient alternative in an emergency situation such as a large-scale radiological accident or nuclear mass casualty scenario for which an easily accessible biosample could be appropriate for high-throughput biodosimetry^17–19^. The great majority of studies dealing with disease biomarkers based on gene expression have been performed using blood or tissue that requires collection by a trained person. With concerted effort, the drawbacks of saliva samples (low RNA yield and high levels of non-human RNA) could be mitigated with methodologic improvements in the laboratory approach.

Our group has extensive experience in the isolation of RNA from whole blood, its cellular subgroups and biopsies of different tissue sources^20–24^. We recently became interested in isolating RNA from saliva. Contradictory results indicated two major challenges:

1. Overwhelming bacterial contamination of the samples.
2. Low abundance of human RNA compared with blood.

In this study, we qualitatively and quantitatively describe these two challenges and how we sought to overcome them. A consistent method for performing gene expression analysis in saliva and dealing with these limitations has never been coherently described before and this work may improve efforts of others in trying to extract RNA in a consistent and well documented way. Herein, we focussed on mRNAs which have shown to be promising biomarkers in other types of body tissues and fluids.

## Materials and methods

### Concept: conventional vs. modified workflow

In order to process human whole saliva to gene expression analysis, we modified several aspects of manufacturer recommendations in the workflow of RNA isolation, cDNA synthesis and Real-Time Quantitative Reverse Transcription Polymerase Chain Reaction (qRT-PCR) in specific ways as described in Table 1. We introduced (1) a cDNA synthesis of only human RNA species, (2) a pre-amplification step and (3) its linearity control. In addition, we isolated whole blood total RNA to compare quality and quantity of extracted RNA to that extracted from human saliva.

**Table 1:** Displayed are the different steps (columns) in gene expression analysis

	RNA extraction	cDNA synthesis	Preamplification	qRT-PCR
**Conventional workflow**				
Kits used	mirVana miRNA Isolation Kit	High-capacity cDNA reverse transcription kit		TaqMan Universal PCR Master Mix
Task	Extraction of total RNA	cDNA synthesis using random primers		qRT-PCR specific for the concerning targets
**Modified workflow**				
Kits used	Oragene protocol + mirVana miRNA Isolation Kit	SuperScript III First-Strand Synthesis SuperMix Kit	Taqman PreAmp Master Mix Kit	TaqMan Universal PCR Master Mix
Task	Extraction of total RNA from whole saliva (human – panbacterial)	cDNA synthesis from human RNA by using oligo(dT) primers	Unbiased and multiplex amplification of up to 100 targets	Unbiased qRT-PCR specific for the concering targets
Rationale	Isolation of RNA from whole saliva	poly(A) + -selected human cDNA	Increase quantity of human cDNA	Unbiased qRT-PCR

Also shown are the comparisons between the conventional workflow (suggested by the manufacturer) and our modified workflow, including the required kits, the tasks and the rationale for our modifications.

The experimental design was granted by the Ethics committee (Bayerische Landesärztekammer, Munich, Germany). The qRT-PCR related measurements (e.g. RNA quantity/quality and TaqMan qRT-PCR) were performed according to the standard operating procedures implemented in our laboratory in 2008 when the Bundeswehr Institute of Radiobiology became DIN-accredited by TÜV Süd München, Germany (DIN EN ISO 9001/2008).

### Sample collection

Whole saliva samples were collected using 4 ml ORAgeneRNA (RE-100) vial collection kits from DNA Genotek according to the manufacturer’s instructions (DNA Genotek Inc., Kanata, Ontario, Canada). The kit is an all-in-one system for the collection, stabilization and transportation of RNA from saliva. Unstimulated whole saliva was collected from twelve healthy donors (5 females, 7 males, average age 32). The self-collection kits are provided with an RNA stabilizing solution to prevent degradation of RNA by RNases. Two ml of saliva was expectorated into the provided vial and mixed with 2 ml stabilizing solution in the lid, making a total volume of 4 ml. The whole saliva was collected from 9 to 10 am and was preserved in the OrageneRNA Self-Collection Kits after having been shaken vigorously. Samples were stored at room temperature overnight and placed in a freezer (− 20 °C or lower) for storage within 1 day.

After completing normal oral hygiene, donors were not allowed to eat or smoke 2 h prior to collection or to drink at least 1 h prior to collection.

As a “positive control”, whole blood samples (2.5 ml) from six of the twelve healthy donors were drawn into PAXgene Blood RNA tubes (Qiagen, PreAnalytiX GmbH, Hilden, Germany) at the Bundeswehr Institute of Radiobiology on the day of saliva collection. The tubes were gently inverted (10 times), kept at room temperature overnight and then stored at − 20 °C.

All samples were anonymized and obtained with informed consent from all donors. Sampling methods were carried out in accordance with the institutional guidelines and regulations.

### RNA extraction

Whole saliva samples were processed following a combination of the ORAgene RNA purification protocol and the mirVana kit protocol (Invitrogen, ThermoFisher Scientific, Carlsbad, CA 92008 USA/Life Technologies, Darmstadt, Germany). Total RNA, i.e. human RNA and pan-bacterial RNA, was isolated. In brief, the frozen (− 20 °C) ORAgene RNA collection tubes were thawed at room temperature.

The first steps were performed according to the *ORAgene RNA purification protocol*^25^: after vigorous shaking, the samples were heated in a 50 °C water bath for one hour in order to homogenize the solution. After taking aliquots of 500 µl (5–6 aliquots per sample) and incubating them at 90 °C for 15 min, samples were cooled to room temperature and 20 µl ORAgene neutralizer solution (1/25 of total volume) was added to each aliquot as buffer and to precipitate the nucleic acids. After incubation on ice for 10 min and centrifugation at 13,000*g* (rcf, relative centrifugal force) for 3 min, the cell-free clear supernatant was pipetted to a fresh Eppendorf tube, while the separated pellet containing the turbid impurities was discarded.

At this step we diverged from the ORAgene RNA purification protocol by skipping the precipitation step with Ethanol and adopted the *mirVana kit protocol*^26^. We added the Lysis/Binding Solution from the mirVana kit. Then total RNA including small RNA species was isolated by combining a Phenol–Chloroform RNA precipitation with further processing and purification using a silica membrane. After several washing procedures to purify RNA from other residual debris, DNA ingredients became digested on the membrane (RNAse free DNAse Set, Qiagen, Hilden, Germany). RNA was eluted with 100 µl RNAse free water in a collection tube and the aliquots were pooled for each sample before freezing at − 20 °C. The filters were discarded, and the tubes were stored until quantitative and qualitative analysis.

The PAXGene tubes containing the whole blood (n = 6) were thawed, washed and centrifuged according to the PAXgene Blood RNA system protocol (BD Diagnostics, PreAnalytiX GmbH, Hombrechtikon, Switzerland). Cells in the supernatant were lysed (Proteinase K; BD Diagnostics, PreAnalytiX GmbH, Hombrechtikon, Switzerland), then the Lysis/Binding Solution was added and further steps were performed according to mirVana kit protocol described above.

Quantity of isolated total RNA from saliva and blood was measured spectrophotometrically using NanoDrop One Microvolume UV–Vis spectrophotometer (NanoDrop, PeqLab Biotechnology, Erlangen, Germany). The results were displayed in ng/µl along with ratio absorbance value at 260 and 280 nm. The ratio of absorbance at 260 and 280 nm (260/280) was used to assess the purity of RNA in the sample. RNA integrity/quality was assessed by the 2100 Agilent Bioanalyzer (Life Science Group, Penzberg, Germany) and DNA contamination was controlled by conventional PCR using a β-actin primer. Taking known quantitative and qualitative baselines from RNA specimens isolated from whole blood, a ratio of A_260_/A_280_ ≥ 2.0 (Nanodrop) and a RNA integrity number (RIN) ≥ 6 would indicate adequate quality of the saliva samples for qRT-PCR analysis.

### Conventional cDNA synthesis—high-capacity cDNA reverse transcription kit

For analyzing gene expression of human rRNA (18S) and pan-bacterial rRNA (16S, see below), total salivary RNA was conventionally converted into complementary DNA (cDNA) via reverse transcription using the High-capacity cDNA reverse transcription kit^27^ (Applied Biosystems, Life Technologies, Darmstadt, Germany). Every single strand of isolated RNA was converted into cDNA via reverse transcription using random primers. The amount of RNA input was always determined to 1 µg. The calculated sample input volume was diluted in RNase free water until a final volume of 50 µl was obtained. Total RNA was reverse transcribed in 100 µl of total reaction mixture containing 10 µl of 10 × RT buffer, 4 µl of 10 mM deoxynucleotide triphosphate (dNTP), 10 µl of random primers, 21 µl of RNase free water and, 5 µl of reverse transcriptase enzyme. Each RNA sample was reverse transcribed using a two-step PCR protocol: 2 h and 15 min in a cycle of 25 °C for 10 min, 37 °C for 2 h and 85 °C for 5 min followed by infinite hold at 8 °C.

Because of the presence of specific abundant transcripts, namely 16S and 18S, the synthesized cDNA was serially diluted using a buffer II (KCl, TWEEN, EDTA, TRIS buffer and RNase free water). Three different cDNA dilutions were prepared out of the stock cDNA solution: 10 ng/10 µl, 0.1 ng/10 µl and, 0.01 ng/10 µl. The concentration with the least amount of cDNA (0.01 ng/10 µl) was used for qRT-PCR amplification of 16S and 18S. The diluted cDNA samples from the High Capacity Kits were stored in − 20 °C until qRT-PCR.

The quantity of synthesized cDNA was measured fluorometrically via Qubit 2.0 Fluorometer (Invitrogen, ThermoFisher, Waltham, Massachusetts, USA) using Qubit ssDNA Assay Kit.

### Modified cDNA synthesis—superscript III first-strand synthesis system

The SuperScript III first-strand synthesis system was newly introduced to synthesize cDNA from the isolated salivary RNA in order to subsequently perform gene expression analysis of human origin. The SuperScript III reverse transcriptase catalyzed reaction utilizes Oligo (dt)_20_ primers which selectively target and amplify the 3′ poly(A) + tail of the vast majority of eukaryotic messenger RNA (mRNA) and thereby is specific for human RNA only. As a result, pan-bacterial RNA will be excluded from further processing. This decreased non-human RNA amplification bias afterwards which is common in the presence of RNAs of non-human origin.

According to the kit description, the amount of starting material can vary from 1 pg to 5 μg of total RNA and the maximum input volume of RNA is 8 µl^28^. In order to increase the amount of input for downstream gene expression analysis, our samples were steamed at 45 °C for 90 min followed by elution with 30 µl of RNase free water. Using the concentration from repeated NanoDrop measurements, the amount of RNA input was defined as 0.5 µg, conforming to the maximum input volume of 8 µl. Each reverse transcription reaction contained 1 µl of Annealing Buffer, 1 µl of 50 µM Oligo(dT)_20_ primer, 10 µl of 2 × First-strand Reaction Mix, 2 µl of SuperScript III/RNaseOUT Enzyme mix and RNase/DNase free water (depending on the existing sample volume to obtain a total of 20 µl). The ingredients were processed in a 0.2 ml PCR tube according to the manufacturer’s instructions in a two-step PCR. Afterwards, the tubes were cooled on ice and stored at − 20 °C until subsequent pre-amplification. To increase the cDNA output, the reactions were performed twice for each sample and later combined into one tube prior to pre-amplification and qRT-PCR.

### Pre-amplification and its control

To detect low abundant RNA species, pre-amplification was required. We added this step as part of our methodologic improvements (see Table 1). We used TaqMan PreAmp Master Mix (Thermo Fisher Scientific Baltics UAB, Vilnius, Lithuania) to increase the amount of up to 100 specific cDNA targets, synthesized with the SuperScript III First-Strand Synthesis System. According to the manufacturer, pre-amplification with this kit is linear and unbiased when a minimum amount of cDNA molecules is present (minimum of 1–250 ng and Ct-values without pre-amplification should be < 35)^29^. Based on the manufacturer, multiplex amplification can be performed by pooling up to 100 TaqMan Gene Expression Assays of interest and combined with cDNA and TaqMan PreAmp Master Mix for pre-amplification PCR^29^. The pre-amplification product was then used for qRT-PCR, using the corresponding TaqMan Gene Expression Assays.

Pre-amplification was performed according to the TaqMan PreAmp master mix kit protocol. In the present work, four different TaqMan Gene Expression Assays (*CDKN1A,* Hs00355782_m1; *FDXR*, Hs01031617_m1; *DDB2*, Hs00772068_m1; and *GAPDH,* Hs00902257_m1) were utilized and pooled to enable the multiplex amplification of specific cDNA targets. Candidate genes were selected based on two criteria: (1) Genes that were known to be expressed in sufficient quantities for gene expression analysis in whole blood and/or (2) genes that were described to be radiation sensitive in previous studies^23,30^. To ensure linearity of the pre-amplification step, the thermal cycling was set to 10 and 14 cycles for each sample in order to show linearity and, thus, an unbiased pre-amplification. For instance, a Ct value of 32 before pre-amplification would result in expected Ct values of 22 and 18 after 10 and 14 cycles of pre-amplification, respectively.

### Real-time quantitative reverse transcription polymerase chain reaction (qRT-PCR)

Using different sets of primers, two kinds of cDNAs were utilized for qRT-PCR: for human (18S rRNA, Hs99999901_g1) and pan-bacterial (16S rRNA, Ba04230899_s1) primer probe designs, cDNA with a low concentration such as 0.01 ng/10 µl from High-capacity cDNA reverse transcription kit was used, whereas for the other primer probe designs (*CDKN1A, FDXR, DDB2* and *GAPDH*, SuperScript III First-Strand Synthesis SuperMix synthesized, i.e. human cDNA with 10 × and 14 × as well as without pre-amplification was used for detection of each of these low abundant genes in each sample (10 ng/10 µl). The qRT-PCR reaction contained TaqMan Universal PCR Master Mix and one of the inventoried TaqMan Gene Expression Assays for separate detection of candidate transcripts. All measurements were run in duplicate. Using a 96-well-forat TaqMan qRT-PCR platform, cards were sealed, centrifuged (1,200 rpm, 1 min, Multifuge3S-R, Heraeus, Germany) and transferred into the ABI PRISM 7900HT sequence detection system. All technical procedures for accurate qRT-PCR were performed in accordance with standard operating procedures implemented in our laboratory in 2008 when the Bundeswehr Institute of Radiobiology became certified according to DIN EN ISO 9001/2008. All chemicals using TaqMan chemistry were provided by Life Technologies, Darmstadt, Germany.

Following qRT-PCR amplification, the 18S/16S rRNA ratio was calculated between raw Ct values of 18S and 16S to compare the quantitative relation of human RNA to bacterial RNA. The fold change was determined using the formula: fold change = 2^ ^(Ct 18S rRNA – Ct 16S rRNA)^.

Furthermore, the observed and expected fold difference of 18S rRNA between 14 and 10 × pre-amplification as well as 14 ×/10 × and unamplified SuperScript III First-Strand Synthesis SuperMix synthesized cDNA was assessed to evaluate a linear and unbiased pre-amplification among the different samples and genes.

### Statistical analysis

The correspondence of the manufacturer’s prediction of a successful pre-amplification (< 35 Ct-values for genes before amplification) and our measurements (proofing the linear amplification using unamplified Ct-values and two amplification rounds) was examined for each gene and comparison using a two-by-two contingency table comprising:

- True positives, TP (manufacturer predicts success and proof of linear amplification was demonstrated),
- True negatives, TN (manufacturer predicts failure and no linear amplification was examined),
- False positives, FP (manufacturer predicts success, but no linear amplification was examined) and
- False negatives, FN (manufacturer predicts failure, but linear amplification could be shown).

To evaluate the predictive ability, we calculated:

- Positive predictive value in percent (PPV, calculated as TPx100/(TP + FP)),
- Negative predictive value in percent (NPV, calculated as TNx100/(TN + FN)),
- FP in percent (FPx100/(TP + TN + FP + FN)),
- FN in percent (FNx100/(TP + TN + FP + FN)).

In this study, a PPV of 100% predicts, that 100% of reactions worked in our measurements when the manufacturer says it should work (according to a Ct value without pre-amplification < 35). A NPV of 100% predicts, that 100% of reactions didn’t work in our measurements when the manufacturer says it shouldn’t work (according to a Ct value without pre-amplification > 35) resulting in an agreement. FP represent the number of samples, in which the pre-amplification should work according to the manufacturer, but our findings (proof of linear pre-amplification) provide insufficient measurements and indicate disagreement. FN represent the number of samples, in which the pre-amplification shouldn’t work according to the manufacturer, but our findings provide reliable measurements, also indicating disagreement.

Excel 2010 (Microsoft) was used for descriptive statistics (n, mean, standard error of mean, standard deviation, min, max) and generating the tables. SigmaPlot Version 14 (Systat Software, Inc., San Jose California USA, https://www.systatsoftware.com) was used for graphical presentations and comparisons among groups (p-values with parametric t-tests or non-parametric tests, where applicable).

## Results

### RNA isolation

**Figure 1:**
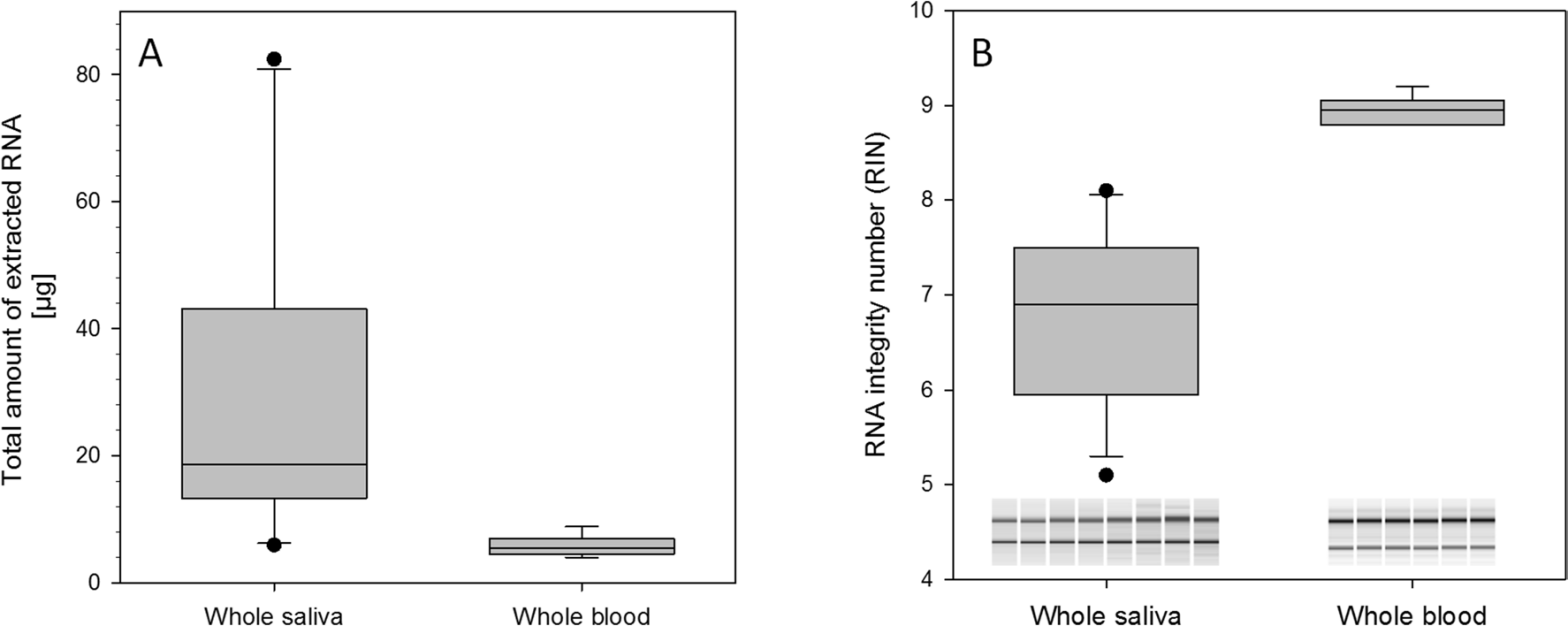
Box plots show the amounts of total RNA (human and bacterial) in µg isolated from whole saliva (2 ml, n = 12) and whole blood (2.5 ml, n = 6) for comparison (**A**). Quality of isolated RNA is shown using RNA integrity numbers (RIN) for saliva samples and blood samples (**B**). Gel-like images created by the Agilent bioanalyzer display bands of 28S and 18S rRNA for eight randomly selected saliva samples and are shown as an inserted picture in (**B**).

We isolated high yields of total RNA from 2 ml saliva samples, resulting in an average of 30.6 µg (SD + /− 19.7). Compared to this, yields of RNA isolated from the 2.5 ml whole blood samples were low (mean 5.8 µg, SD + /− 1.3, Fig. 1A). Moreover, high purity RNA with OD_260/280_ ratios at a mean of 2.1 was isolated from saliva. A mean RNA integrity number (RIN) of 6.7 (SD + /− 0.8) was detected for saliva samples using the Agilent bioanalyzer and all saliva samples showed gel-like image bands of 28S and 18S that did not provide any indications for severe degradation (Fig. 1B). The mean RIN for PAXGene whole blood samples was 9.0 (SD + /− 0.1), suggesting high quality RNA. No DNA contamination could be detected in all samples (data not shown).

### Measurement of 18S rRNA Ct values—human RNA only

**Figure 2:**
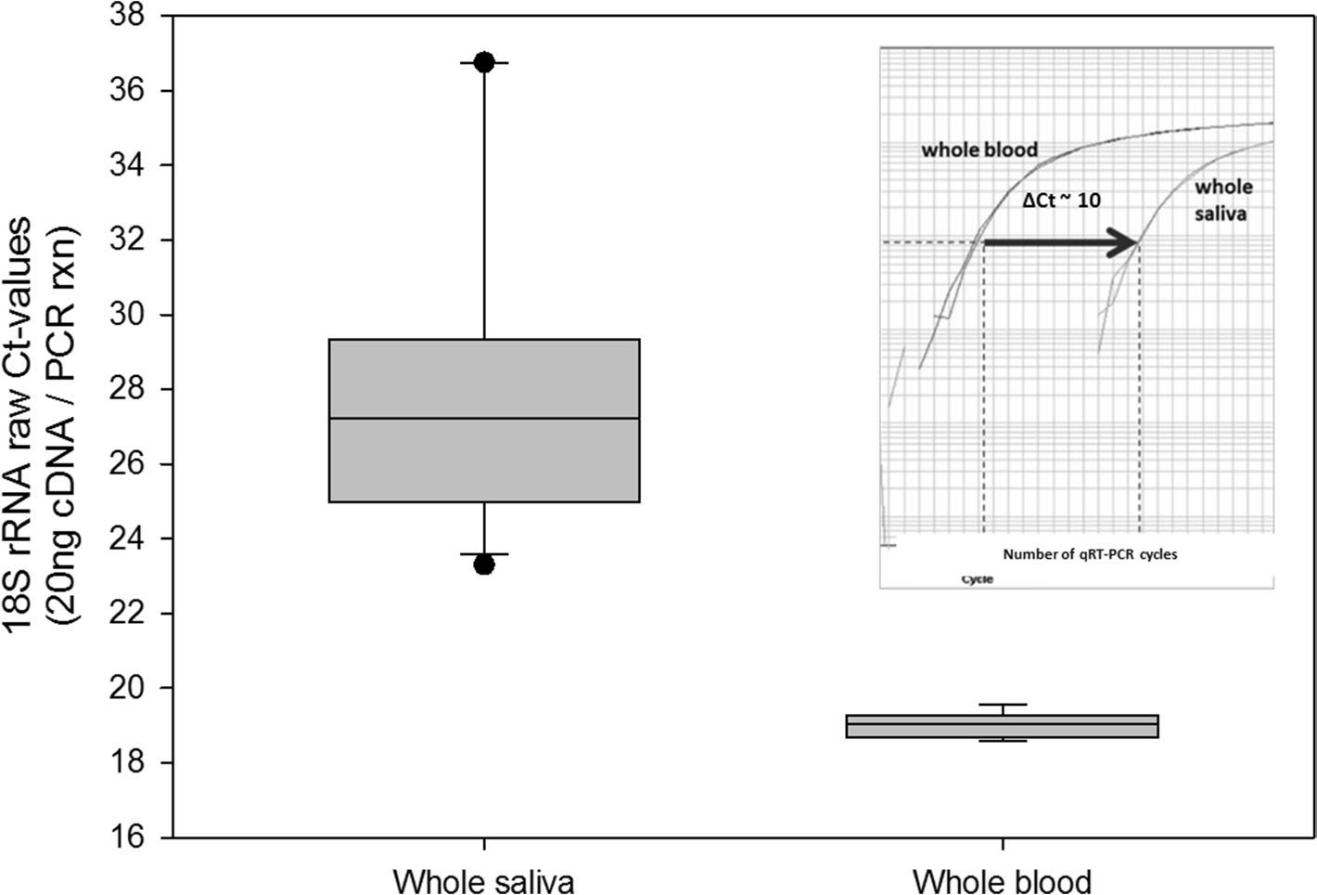
The box plots display the 18S rRNA raw Ct-values for both whole saliva (n = 12) and whole blood (n = 6) samples. Of note, the input amount for cDNA synthesis for each sample was 1 µg and the amount of cDNA input for qRT-PCR was 20 ng—regardless of whether saliva or blood was used. For an example, amplification plots for 18S rRNA are shown for one saliva sample and the corresponding blood sample as an inserted figure. Droplines (vertical dashed lines) provide the corresponding cycles required for the fluorescent signal to cross the threshold (Ct values). Ct values for detection of human 18S rRNA show a Ct-difference of about 10 Ct-values corresponding to 2^ ^10^ ~ 1,000-fold differences in RNA copy numbers.

Human eukaryotic 18S rRNA raw Ct-values were measured for both saliva and blood samples. Using the same quantity of total RNA (0.02 µg total RNA/qRT-PCR reaction) in saliva samples, the mean human 18S rRNA Ct value was 28.1 with large fluctuations (SD + /− 4.2, min 23.3, max 36.8). In blood samples, mean 18S rRNA Ct values of 19.0 could be detected with low fluctuations (SD + /− 0.28, min 18.6, max 19.6) using the same amount of total RNA per reaction (Fig. 2). Comparing the mean 18S rRNA Ct values and taking into consideration that 18S rRNA is representative for most human RNA copy numbers, there was 2^ ^(28.^^1–19^^.0)^ = 549 times less human RNA in saliva compared to blood.

Hence, similar quantities of total RNA input for saliva and blood RNA samples resulted in about 549 times lower human 18S rRNA copy numbers in saliva compared to 18S rRNA measurements in whole blood.

These findings led us to the following questions:

1. Was there an inhibition of cDNA synthesis?
2. Was there an inhibition of qRT-PCR?
3. Was there significant bacterial contamination?

### Addressing inhibition of cDNA-synthesis

In order to exclude an inhibition of cDNA synthesis caused by residual contaminants in collected whole saliva, we measured the quantity of cDNA synthesized with the high-capacity cDNA reverse transcription kit fluorometrically via Qubit 2.0 Fluorometer using Qubit ssDNA Assay Kit. This showed a total average amount of salivary cDNA of 0.36 µg (SD + /− 0.14, min 0.2, max 0.76) and 0.47 µg (SD + /− 0.17, min 0.24, max 0.69) for cDNA from blood samples (supplement Fig. 1A), which was not statistically different from each other. This observation does not imply inhibition of cDNA synthesis.

### Addressing inhibition of qRT-PCR

To detect if there was an inhibition of qRT-PCR, a dilution series of cDNA synthesized via high-capacity cDNA reverse transcription kit from saliva and blood was performed, resulting in cDNA concentrations from 0.01 to 0.00001 ng/10 µl (four decimal steps). Eukaryotic (18S) rRNA raw Ct-values were measured via qRT-PCR and are graphically shown in supplemental Fig. 1B. PCR efficiency was close to 100% and the slopes were not statistically significantly different for 18S Ct values below 35 (mean 3.52, SD + /− 0.015 for saliva compared to mean 3.58, SD + /− 0.01 for blood, P = 0.333). 18S rRNA raw Ct-values differed for saliva and blood by about 8 Ct values. But this shift was similar for all dilutions with 18S rRNA Ct values below 35, which means that linearity between blood and saliva persists in a range below Ct values of about 35. Above 35, curves tend to reach a saturation point and linearity disappears because amounts of input for qRT-PCR are too low. These results clearly suggest that there is no inhibition of qRT-PCR when using human salivary material.

### Measurement of 16S rRNA Ct values—ratio of human to bacterial RNA

Considering that total RNA amounts measured with NanoDrop were in a similar range for blood and saliva (regardless of the large fluctuations in salivary RNA), this strongly suggested that large amounts of salivary RNA isolated originated from bacteria. For this reason, pan-bacterial 16S rRNA raw Ct-values were measured for whole saliva, showing a mean of 17.2 (SD + /− 0.5, min 16.6, max 18.2) and indicated that there are high yields of bacterial RNA in the sample. The mean ΔCt 18S/16S was 10.9 with a maximum of up to 19.8 (SD + /− 4.2, min 5.4, max 19.8). Calculating the fold change between human and bacterial rRNA (2^^ΔCt^), this resulted in a mean 18S/16S-rRNA-ratio, i.e. degree of bacterial contamination, of 1:110,465. This means that for each copy of a human gene, on average 110,465-times more copies of bacterial genes can be found in the samples, taking again into consideration, that 18S rRNA and 16S rRNA, as the most expressed housekeeping genes, are representative for the majority of human and bacterial RNAs. Depending on the 18S/16S-rRNA-ratio for each sample, the degree of bacterial contamination can range from 42 to more than 900,000 (Table 2). These results quantitatively show a high bacterial contamination in saliva samples.

**Table 2:** Quantification of the human (18S) and pan-bacterial (16S) gene expression measurements (Ct values) as well as the ΔCt 18S-16S and the calculated 18S/16S-RNA-ratios (2^^ΔCt^), i.e. degree of contamination.

Sample ID	18S Ct	16S Ct	∆ Ct 18S -16S	degree of contamination (18S/16S-RNA-ratio)
1	23.3	17.9	5.4	42
2	24.2	17.9	6.3	78
3	25.1	17.4	7.7	204
4	24.9	16.9	8.1	271
5	27.0	17.0	10.1	1,060
6	27.0	16.9	10.1	1,113
7	27.4	17.2	10.2	1,176
8	27.5	16.6	10.9	1,965
9	27.8	16.8	11.0	2,020
10	29.9	17.1	12.8	7,033
11	36.8	18.2	18.6	386,471
12	36.7	16.9	19.8	925,581
Mean	28.1	17.2	10.9	1,145
Min	23.3	16.6	5.4	42
Max	36.8	18.2	19.8	925,581
SD	4.2	0.5	4.2	267,609
SEM	1.2	0.1	1.2	77,252

12 Samples are sorted in ascending order for the calculated 18S/16S-RNA-ratios. The table below the horizontal double bar aggregates the 12 data sets for 18S and 16S Ct values, ΔCt 18S-16S as well as the 18S/16S-RNA-ratios and provides descriptive statistics: mean, min (minimum), max (maximum), SD (standard deviation) and SEM (standard error of the mean).

### qRT-PCR—“unbiased” gene expression measurements?

As described above, our aim was to validate the expression stability and unbiased amplification of four selected genes to show an improvement in laboratory technique. Four genes (*CDKN1A, FDXR, DDB2* and *GAPDH*) were detected in 10 out of 12 the saliva samples after 10 × and 14 × pre-amplification. In two samples (sample ID 11 and 12), no amplification plots could be determined when not pre-amplified and no reliable amplification plots, i.e. Ct values, could be determined when pre-amplified, both after 10 × and 14 × pre-amplification (data not shown). These samples showed the lowest amounts of human RNA and the highest bacterial contamination (Table 2). For the rest of the samples (n = 10), Ct values were determined in all samples and for all genes after 10 × and 14 × pre-amplification and in the vast majority of the non-pre-amplified samples depending on the gene (supplemental Table 1).

**Figure 3:**
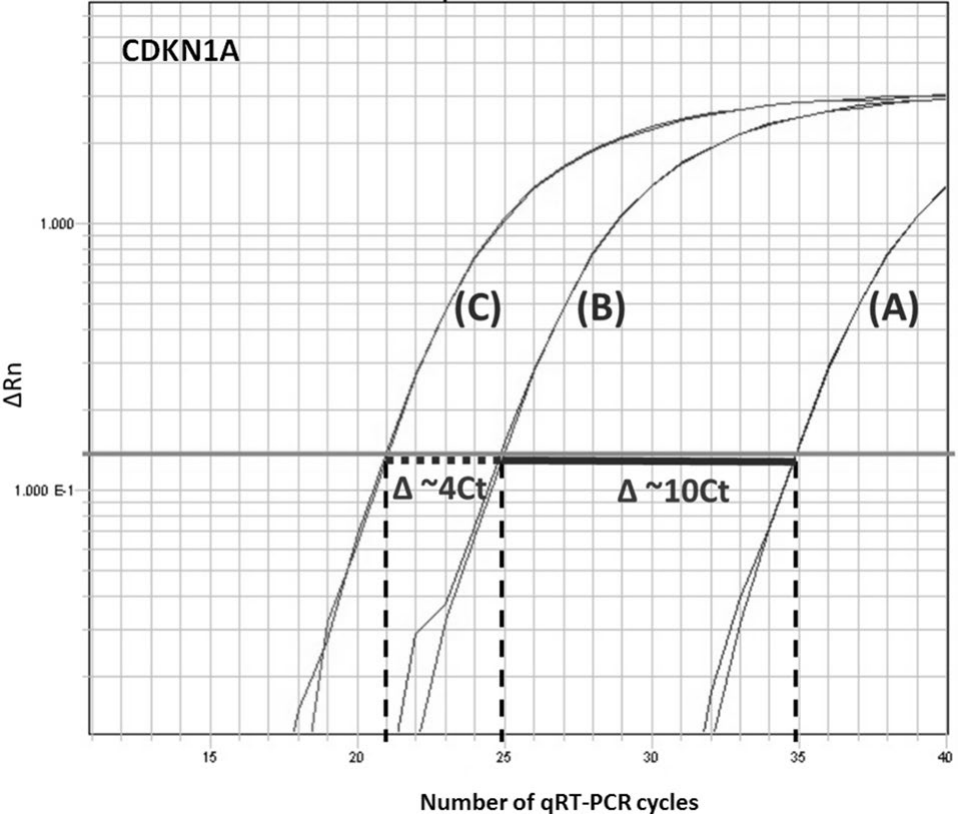
The figure depicts three exemplary amplification plots (unamplified, 10 × and 14 × pre-amplified) from one saliva sample (sample ID 7) and one gene (*CDKN1A*) created with the ABI PRISM 7900HT sequence detection system. The amplification plot (**A**) represents the unamplified sample, the one marked with (**B**) the 10 × pre-amplified sample and the one with (**C**) the 14 × pre-amplified sample. Droplines (vertical dashed lines) provide the corresponding cycles required for the fluorescent signal to cross the threshold (Ct values). Linearity of 10 × pre-amplification is shown as a ΔCt of 10 in horizontal solid line 1 (Ct _(no-Amp)_—Ct _(14X pre-Amp)_), and corresponding ΔCt of 4 in horizontal dotted line 2 (Ct _(10X pre-Amp)_—Ct _(14X pre-Amp)_). ΔRn (normalized reporter value): the Rn value of an experimental reaction minus the Rn value of the baseline signal.

After pre-amplification and qRT-qPCR, amplification linearity between unamplified and 10 ×/14 × pre-amplified PCR products of the four genes was calculated by using the following formula: Ct _(no-Amp)_ − Ct _(14X pre-Amp)_. A ΔCt of 10, respectively 14 indicates 100% linearity and efficiency of the pre-amplification of the target gene. *CDKN1A* was found to be consistently expressed in all remaining samples with a mean Ct value without pre-amplification of 33.1, 23.2 after 10 × and 19.3 after 14 × pre-amplification (Fig. 3). This implies that after pre-amplification, best results were observed with Ct values closest to the expected Ct values (Table 3). A mean ΔCt _(observed vs expected)_ was calculated, resulting in 0.09 for 10 × pre-amplified samples and 0.24 for 14 × pre-amplified samples. Mean ΔCt values are shown in Table 3 and single Ct values are depicted in supplemental Table 1. The corresponding Δ can be seen in an acceptable range for being caused by methodological issues. This means that pre-amplification and qRT-PCR was unbiased with best linearity and stability for *CDKN1A*. Ct values after pre-amplification were in a range that is totally acceptable for gene expression analysis.

**Table 3:** The table summarizes qRT-PCR results (mean raw threshold cycle [Ct]-values) of four gene targets (*CDKN1A, FDXR, DDB2* and *GAPDH*) for the saliva samples.

Genes	Pre-amplification	Measurements				Interpretation		
		# Samples/comparisons	Mean Ct values		Difference observed vs expected	Manufacturer predicts success	Our findings: works?	Agreement?
			Observed	Expected				
*CDKN1A*	0 ×	12	33.1			Yes	Yes	Yes
	10 ×	12/12	23.2	23.1	0.09			
	14 ×	12/12	19.3	19.1	0.24			
*DDB2*	0 ×	12	36.1			No	No	
	10 ×	12/12	27.9	26.1	1.8			
	14 ×	12/12	24.5	22.1	2.33			
*FDXR*	0 ×	12	37.4			No	Yes	No
	10 ×	12/12	28.4	27.4	1.02			
	14 ×	12/5	23.9	23.4	0.49			
*GAPDH*	0 ×	12	33.5			Yes	No	
	10 ×	12/12	29.3	23.5	5.78			
	14 ×	12/12	26.2	19.5	6.69			
In total		**12/89**						

Number of samples and number of comparisons per gene and pre-amplification pattern are depicted in italics. Shown are the measured/observed mean Ct values for unamplified samples as well as for samples after 10 × and 14 × pre-amplification. The column for expected mean Ct values shows the calculated Ct values when estimating a 100% linear and unbiased 10 × and 14 × pre-amplification (steps by 10 and 14, taking the mean Ct value without pre-amplification). The ΔCt _(observed vs expected)_ is shown for each gene and each pre-amplification. We categorized the data by whether unbiased pre-amplification should have worked according to the manufacturer (Ct value without pre-amplification < 35, yes) or not (Ct value without pre-amplification > 35, no). The data are further presented if pre-amplification worked unbiased (yes) or not (no) according to the amplification biases through deviating expression patterns between amplified and un-amplified material.

**Figure 4:**
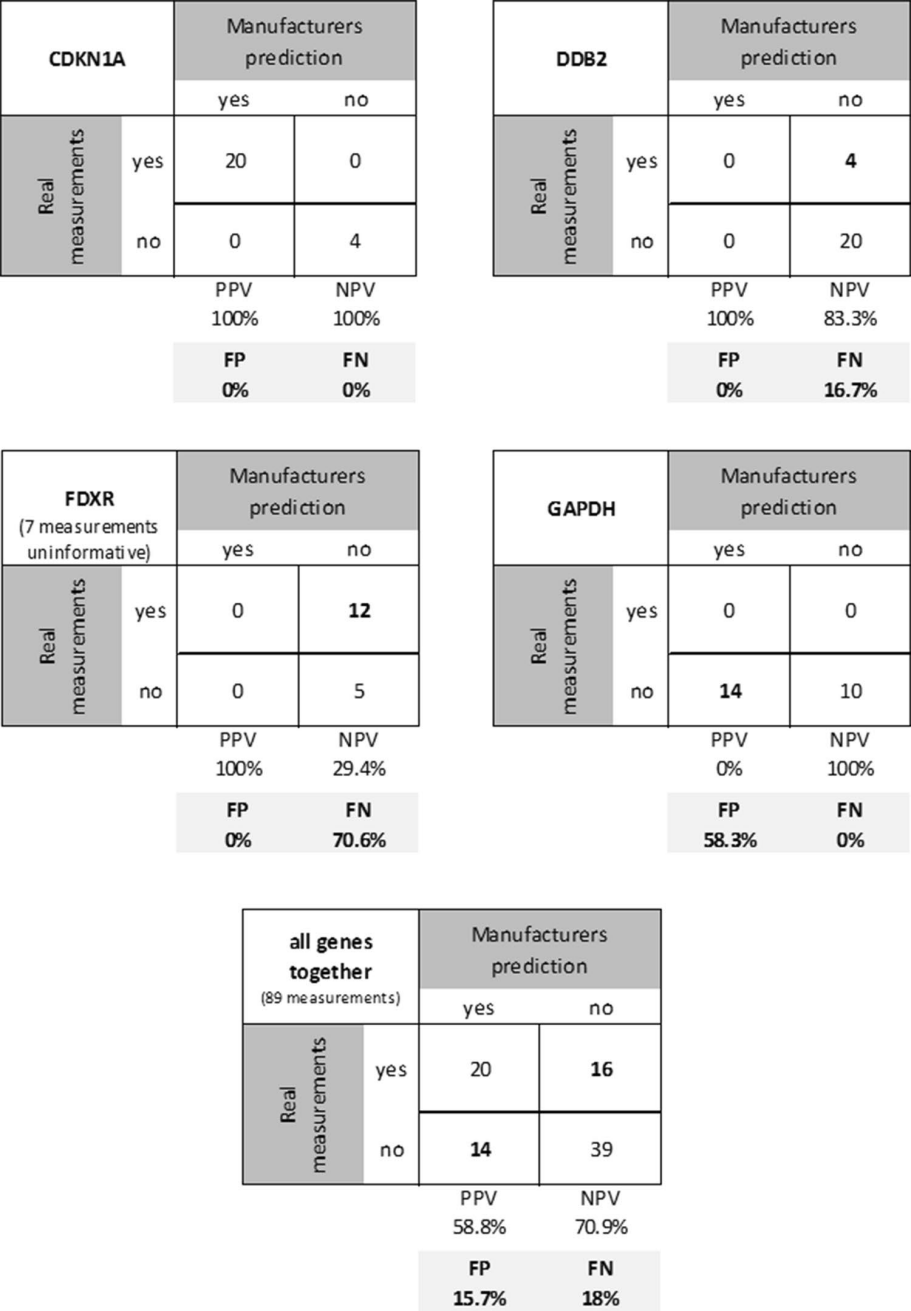
The figure depicts a two-by-two contingency table for each gene and for all genes together in order to statistically quantify the correspondence of the manufacturer’s prediction of a successful pre-amplification and our measurements. Numbers in bold represent the false negatives (FN, manufacturer predicts failure, but linear amplification could be shown), respectively false positives (FP). Fields in grey depict the values for FP and FN in percent. PPV, positive predictive value; NPV, negative predictive value.

Considering, that according to the manufacturer, a minimum Ct value of 35 is necessary for unbiased pre-amplification, we observed different patterns (Table 3, Fig. 4):

For *CDKN1A* (Ct value without pre-amplification < 35: best results, obviously unbiased) our findings are consistent with the manufacturer (PPV 100%, NPV 100%, FP 0%, FN 0%).

In *FDXR* our results were satisfying regarding Ct values after pre-amplification (mean Ct of 23.9 after 14 ×), whereas, with a mean Ct value of 37.4 without pre-amplification and a majority of non-pre-amplified samples being undetermined during qRT-PCR, the results should be biased according to the manufacturer. This shows a high number of false negatives, respectively 70.6% of all comparisons, which indicates that pre-amplification is linear although it shouldn’t work according to the manufacturer. There are again no false positives.

For *DDB2* the corresponding mean ΔCt _(observed vs expected)_ was 1.8 after 10 × pre-amplification, respectively 2.33 after 14 × pre-amplification (Table 3). This means that linearity of pre-amplification was suboptimal compared to *CDKN1A*. With a mean Ct value without pre-amplification of 36.1, pre-amplification shouldn’t work according to the manufacturer. Although this was true for the majority of comparisons, we found false negative results in 16.6% (PPV 100%, NPV 83.3%, FP 0%, FN 16.7%; Ct value without pre-amplification > 35: limitations, obviously biased).

We found a different pattern for *GAPDH*: while the mean raw Ct value without pre-amplification was below the recommended 35 cycles (33.5) compared to *DDB2*, indicating enough RNA copy numbers for an unbiased pre-amplification and qRT-PCR, the corresponding ΔCt _(observed vs expected)_ was 5.78 after 10 × pre-amplification and 6.69 after 14 × pre-amplification. These outcomes are biased and indicate that the pre-amplification results in an almost 2^^6.69^ = 103 times difference compared to the expected values. This dysregulation results in a high number of false positives (58.3%), whereas no false negatives were found. Hence, the recommended Ct values of target genes below 35 before pre-amplification proved to be not sufficient of itself and had to be augmented by the criteria of showing a linear (unbiased) pre-amplification.

Taken together, predictions based on the manufacturer’s criteria are biased and in our examinations can result in as much as 58.3% false positive and up to 70.6% false negative results (Fig. 4). Hence, based on the manufacturer’s criteria, a considerable number of samples and genes could not be used for analysis. But according to our measurements checking uniformity of pre-amplification, they can be used when following our newly introduced criteria (proofing linearity of the pre-amplification step).

## Discussion

There are numerous advantages to use human saliva biosamples, as described above. The main benefit is saliva is very non-invasive and easy to access for diagnostic and monitoring approaches. Its potential has been shown in numerous studies examining different salivary biomarkers, e.g. in oral diseases like cancer^5,12^.

There are a couple of studies dealing with gene expression profiling in human saliva^14,31,32^, but the method behind has never been explicitly described in detail before, even less described were the challenges, that have to be coped with when working with saliva. Our aim was to provide a robust method to process whole saliva for downstream gene expression studies.

Saliva was not a sterile medium compared to blood or other body tissues. Due to the complex contamination of human whole saliva, RNA from this medium is extremely sensitive to rapid degradation of salivary RNA. Endonucleases like RNAses mainly contribute to that. Nevertheless, we showed that, despite these difficult conditions, the RNA integrity is sufficiently well in order to process human salivary RNA for downstream gene expression analysis using the modified workflow. Or in other words: the salivary transcriptome is stable enough.

In the course of this study we found that human 18S rRNA abundance compared to blood was several log-fold lower as would be expected considering that the same quantity of total RNA was used. Therefore, we approached details step by step supposing that sufficient amounts of high-quality RNA can be extracted from saliva samples and that there is no inhibition of cDNA synthesis or qRT-PCR (Fig. 1 and supplemental Fig. 1). On closer inspection at the composition of total RNA that was extracted from whole saliva, the crucial point could be quantitatively shown: beside the low yields of human RNA, which is the minor fraction of the total RNA, bacterial RNA originating from the oral microbiome posed the vast majority (Table 2).

Therefore, two challenges had to be addressed within our effort:

1. How to deal with the overwhelming bacterial contamination of the samples?
2. How to deal with the low abundance of human RNA, in order to detect gene expression changes of human origin only?

The bacterial contamination, which had a high inter-individual variance, actually posed the main problem when working with whole saliva. We tried to overcome these problems by focusing on the poly(A)+-tails of human RNA using only Oligo (dt)_20_ primers for cDNA synthesis and were able to amplify specific human RNA. But still having to deal with the low yields of material from human eukaryotic origin, downstream pre-amplification became indispensable in the adapted methods we devised to obtain reliable results in downstream gene expression analysis.

Even so, it became obvious to us that this might not apply for all genes: according to the manufacturer of the TaqMan PreAmp Master Mix (Applied Biosystems), pre-amplification is uniform when Ct values without pre-amplification are < 35. In order to identify amplification biases, we introduced two rounds of pre-amplification and examined for a linear amplification among unamplified and 10 × and 14 × pre-amplification rounds. Doing that, we identified those 16.7–70.6% of samples which could not be analysed based on the manufacturer’s criteria (false negatives), but which could be used for analysis following our newly introduced criteria (check uniformity of pre-amplification; Table 3, Fig. 4).

Our findings reliably suggest that pre-amplification achieved excellent results for the majority of samples. But it is not suitable for all genes and an amplification generated bias has to be expected for genes like *DDB2* which is most likely caused by the low copy number in the source material (Ct values without pre-amplification > 35). For *GAPDH* Ct values < 35 before pre-amplification suggest a different reason, which might be the formation of dimers. Pre-amplification should be linear according to the manufacturer but our findings show 58.3% of false positives. Of note, according to the manufacturer, up to 100 TaqMan Gene Expression Assays can be pooled for specific amplification of these targets and directly compare pre-amplified samples without introducing bias^29^. This further supports augmentation of the recommended Ct value of target genes below 35 before pre-amplification by the criteria of showing a linear (unbiased) pre-amplification for each target gene.

Nevertheless, the criteria of Ct values < 35 before pre-amplification appeared important but not primarily decisive (see false negatives for *FDXR*). For instance, the suboptimal pre-amplification (∆Ct observed vs expected) in *DDB2* (compared to *CDKN1A*) is most likely due to the fact that Ct values without pre-amplification were already > 35, whereas *CDKN1A* was < 35 and showed best results (confirming the manufacturer’s specifications). The observed and expected Ct-values for *CDKN1A* are very close and represent a variance as accepted for technical replicate measurements (< 0.5 Ct-values difference). These findings suggest that a minimum amount of input is required such that uniformity of pre-amplification should always be checked. In the scenario described above, one solution was to synthesize more cDNA from human RNA with the SuperScript kit in order to increase the input for pre-amplification and qRT-PCR.

Hence, we demonstrated that in 17–71% of the comparisons, a false prediction is made when considering the Ct-value > 35 in un-amplified samples only. This means that gene expression analysis works in 17–71% of the samples with Ct values above 35, even when the manufacturer maintains that it doesn’t work (false negatives). On the other hand, pre-amplification doesn’t work in 58.3% of certain gene measurements, although manufacturer predicts success. Furthermore, an improved outcome was achieved if pre-amplification showed a linear (unbiased) relation and that it is necessary to check the linearity.

Although the current study was based on a moderate size of samples (n = 12) and gene targets (n = 4), we performed gene expression analysis for each sample and each of the 4 gene targets without pre-amplification as well as after 10 × pre-amplification and 14 × pre-amplification (total of 3 measurements per sample and gene) in order to show linearity and, thus, an unbiased pre-amplification. This comes up to a total of 144 measurements, respectively 96 comparisons. Regarding the independently performed pre-amplification reactions and following qRT-PCRs, a total of 24 reactions (12 samples, 10 × and 14 ×) were performed for each gene (supplemental Table 1). In our opinion, a total number of 24 pre-amplification comparisons per gene (regardless technical replicates) is sufficient for the generation of our hypothesis, which is underlined by the statistics employed (e.g. Fig. 4). By taking these exemplary genes and checking the linearity individually, we are able to show different patterns of disagreement described above: false positives and false negatives.

Due to the moderate sample size we judge our study more as a “proof of concept” study where we explore the potential of mRNAs in human whole saliva. Nevertheless, even increasing the sample size will not alter the three newly introduced modifications which are: (1) Selective cDNA synthesis of mRNA species of human origin, (2) pre-amplification before qRT-PCR and (3) check uniformity of pre-amplification. These are prerequisites for successful future efforts with respect to processing human RNA for biomarker studies, we believe.

For example, 18S rRNA is not synthesized to cDNA when using the SuperScript III First-Strand Synthesis System, because 18S rRNA -transcripts lack a poly(A)+-tail. That’s why 18S rRNA, as a commonly known housekeeping gene, cannot be used as a normalizer in gene expression analysis in the current application. A new housekeeping gene except for rRNA needs to be detected when working on gene expression analysis in human whole saliva.

Second, the composition of whole saliva may be influenced by extraneous factors which may limit its use as a biomarker: demographic characteristics (like age, gender, race), social habits (like smoking, alcohol, diet) or oral hygiene are among the factors that can interfere with the composition of saliva at the time of collection^19^. Further investigations will show if this poses a limitation of its use as a biomarker.

We decided to use whole saliva as a source for RNA biomarkers instead of salivary supernatant due to the numerous advantages described above. Admittedly, a higher background due to a high amount of non-human RNA is a drawback but using whole saliva is much easier than harvesting supernatant and isolating exosomes. Until now, it is not completely clear, whether the oral microbiome alters gene expression patterns in the salivary glands and to what extend this affects the robustness of salivary transcriptomics as a diagnostic tool, e.g. considering gland disorders. Rather, we chose a robust, less elaborate and high through-put approach with high utility for future studies.

In summary, we report a robust modified methodology to process human whole saliva as a non-invasive and easily accessible biofluid for gene expression analysis. These three modifications, described here will be important to overcome the two challenges of an overwhelming bacterial contamination and low abundance of human RNA in the source material for future applications. We suggest that modifications such as ours will render human saliva as a more attractive material for an expanded number of biomarker studies.

## Supplementary information

Supplementary information.

## Data Availability

The datasets generated and analysed during the present study are available from the corresponding author upon request.
