# Supplementary material for: Overcoming challenges in human saliva gene expression measurements
Source: Sci Rep. 2020 Jul 7;10:11147. doi: 10.1038/s41598-020-67825-6 (PMC7341869; doi:10.1038/s41598-020-67825-6)
Supplement: Supplementary file 1 — Supplementary information. [file 41598_2020_67825_MOESM1_ESM.pdf]

Overcoming challenges in human saliva gene expression measurements

Ostheim P[1,*], Tichý A[2], Sirak I[3], Davidkova M[4], Markova Stastna M[5], Kultova G[2], Paunesku T[6], Woloschak G[6], Majewski M[1,7], Port M[1], Abend M[1]

[1] Bundeswehr Institute of Radiobiology, Munich Germany
[2] Department of Radiobiology, Faculty of Military Health Sciences in Hradec Kralove, University of Defence in Brno, Czech Republic
[3] Department of Oncology and Radiotherapy University Hospital and Faculty of Medicine, Hradec Kralove, Czech Republic
[4] Department of Radiation Dosimetry, Nuclear Physics Institute of the Czech Academy of Sciences, Prague, Czech Republic
[5] Institute for Hematology and Blood Transfusion, Hospital Na Bulovce, Prague, Czech Republic
[6] Department of Radiation Oncology, Northwestern University, Chicago, Illinois 60611, United States
[7] Department of Urology, Bundeswehrkrankenhaus Ulm, Germany

Second Revised version
submitted to
Scientific Reports
2nd June 2020

Number of figures: 4 (+2 supplement)
Number of tables: 3 (+1 supplement)

* Corresponding Author: Patrick Ostheim,
Bundeswehr Institute of Radiobiology affiliated to the University of Ulm,
Neuherbergstr. 11, 80937 Munich, Germany, phone/fax: +49-89-992692-2283/-2250,
e-mail: patrickostheim@bundeswehr.org

This work was supported by the German Ministry of Defence

Supplemental figure 1

Ostheim et al.

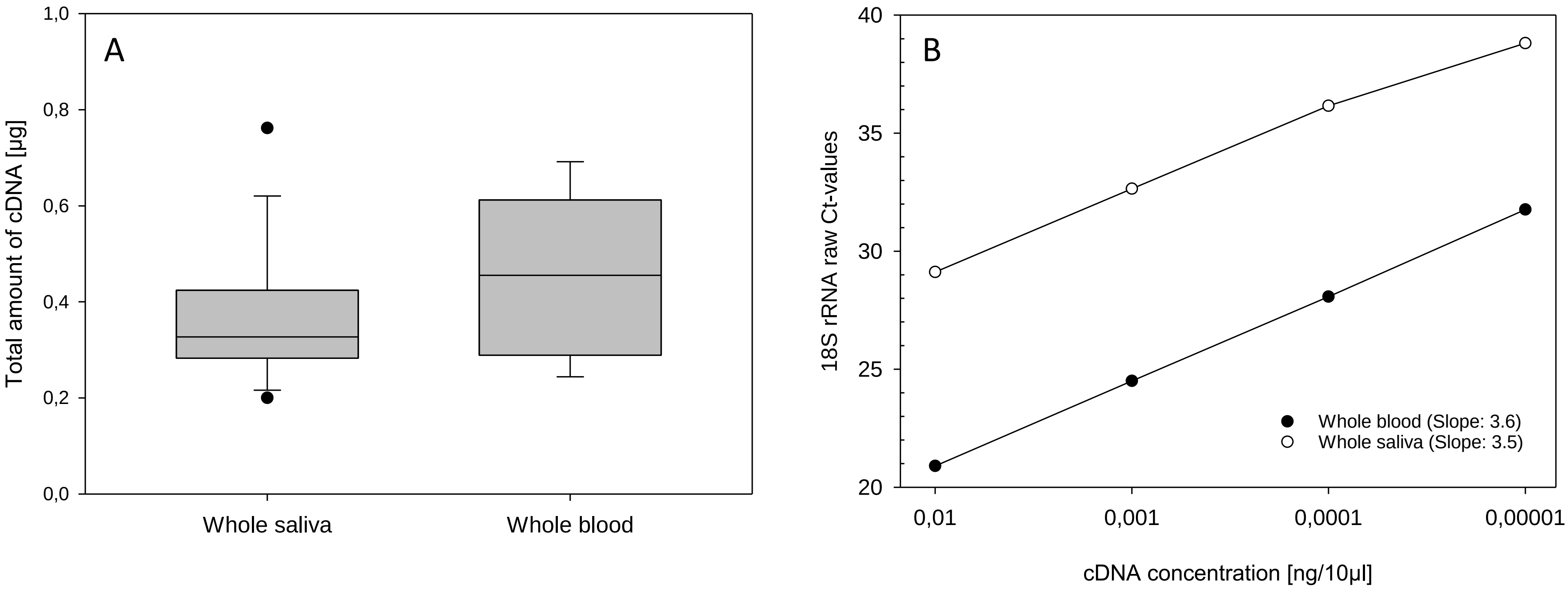

Supplemental figure 1

Box plots in (A) illustrate the total cDNA amounts (µg) measured fluorometrically (via Qubit 2.0 Fluorometer) from whole saliva (n=12) and whole blood (n=6). Input of 1 µg of total RNA was synthesized during cDNA synthesis previously.

Scatter plots in (B) display the results from cDNA dilution series and measurement of 18S rRNA raw Ct-values from whole saliva and whole blood samples using four different cDNA serial concentrations from 0.01 to 0.00001 ng/10µl. Slopes were calculated from the first-order linear regression analysis.

Supplemental figure 2
Ostheim et al.

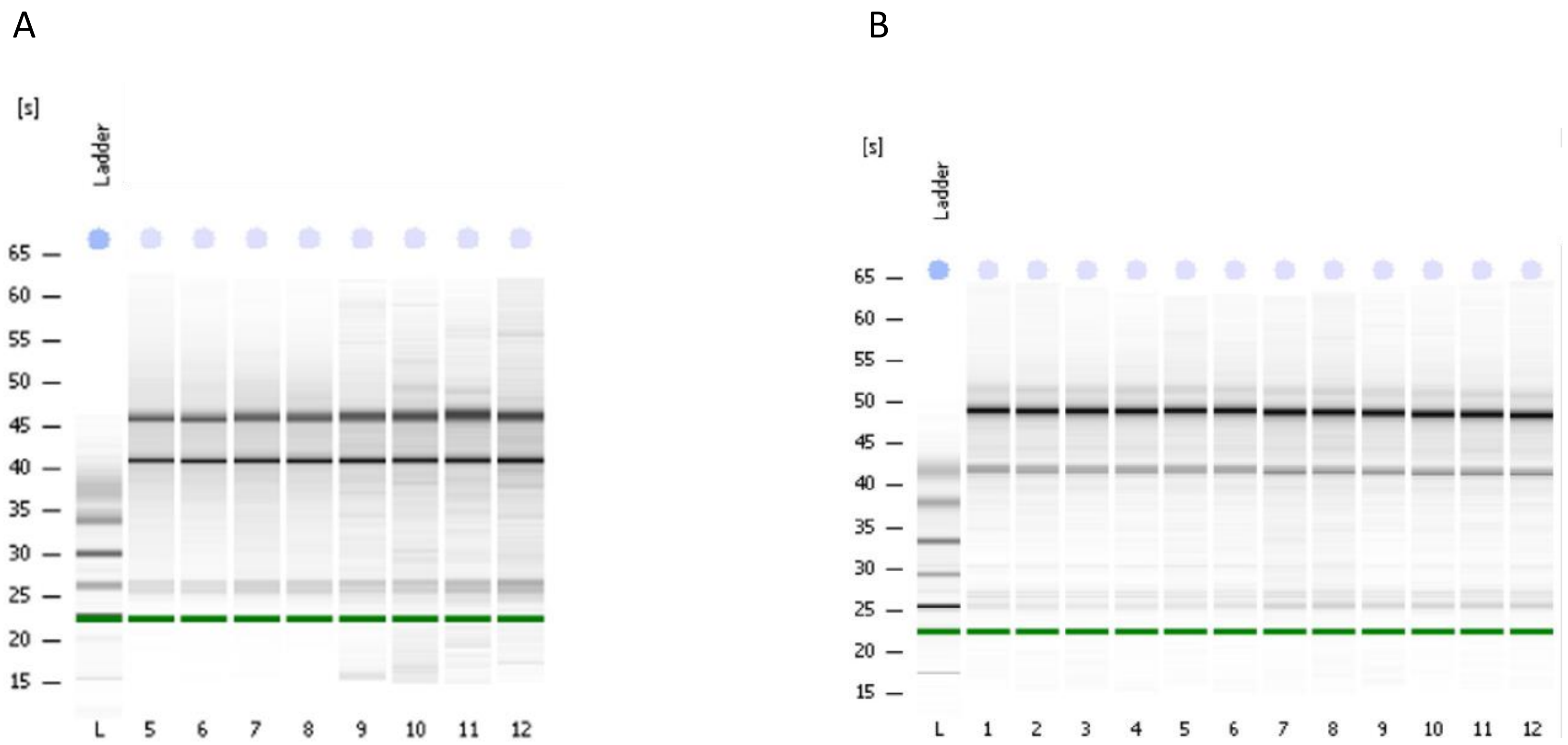

Supplemental figure 2
These Gel-like images created by the Agilent bioanalyzer show the quality of isolated RNA and display bands of rRNA for eight randomly selected saliva samples as well as for six whole blood samples (duplicate measurements). They additionally depict the original versions of the cropped images of figure 1.

Supplemental table 1

Ostheim et al.

Gene	Sample ID	Pre-amplification	Measurements: Mean Ct values observed	Measurements: Mean Ct values expected	Difference observed vs expected	Interpretation: Manufacturer predicts success	Interpretation: Our findings: works?	Interpretation: Agreement?
CDKN1A	***1***	***0x***	**31.3**					
		10x	**21.3**	21.3	-0.02	yes	yes	yes
		14x	**17.5**	17.3	0.20	yes	yes	yes
	2	***0x***	**30.6**					
		10x	**20.6**	20.6	-0.04	yes	yes	yes
		14x	**16.9**	16.6	0.26	yes	yes	yes
	3	***0x***	**33.0**					
		10x	**22.9**	23.0	-0.13	yes	yes	yes
		14x	**19.0**	19.0	0.01	yes	yes	yes
	4	***0x***	**31.5**					
		10x	**21.5**	21.5	-0.02	yes	yes	yes
		14x	**17.8**	17.5	0.31	yes	yes	yes
	5	***0x***	**33.9**					
		10x	**24.5**	23.9	0.54	yes	yes	yes
		14x	**20.5**	19.9	0.59	yes	yes	yes
	6	***0x***	**32.6**					
		10x	**22.6**	22.6	0.03	yes	yes	yes
		14x	**18.7**	18.6	0.12	yes	yes	yes
	7	***0x***	**33.1**					
		10x	**23.3**	23.1	0.25	yes	yes	yes
		14x	**19.7**	19.1	0.57	yes	yes	yes
	8	***0x***	**34.9**					
		10x	**24.7**	24.9	-0.25	yes	yes	yes
		14x	**20.8**	20.9	-0.15	yes	yes	yes
	9	***0x***	**34.9**					
		10x	**25.3**	24.9	0.43	yes	yes	yes
		14x	**21.0**	20.9	0.11	yes	yes	yes
	10	***0x***	**35.0**					
		10x	**25.2**	25.0	0.14	yes	yes	yes
		14x	**21.3**	21.0	0.33	yes	yes	yes
	11	***0x***	**n/a**					
		10x	**34.2**	n/a	n/a	no	no	yes
		14x	**27.6**	n/a	n/a	no	no	yes
	12	***0x***	**n/a**					
		10x	**32.6**	n/a	n/a	no	no	yes
		14x	**27.3**	n/a	n/a	no	no	yes

CDKN1A	***mean***	***0x***	**33.1**					
		10x	**23.2**	23.1	0.09	yes	yes	yes
		14x	**19.3**	19.1	0.24			

Gene	Sample ID	Pre-amplification	Measurements			Interpretation		
			Mean Ct values		Difference observed vs expected	Manufacturer predicts success	Our findings: works?	Agreement?
			observed	expected				
DDB2	1	0x	35.8					
		10x	26.7	25.8	0.97	no	no	yes
		14x	22.9	21.8	1.17	no	no	yes
	2	0x	36.1					
		10x	26.3	26.1	0.20	no	yes	no
		14x	22.6	22.1	0.52	no	yes	no
	3	0x	36.5					
		10x	27.7	26.5	1.20	no	no	yes
		14x	24.1	22.5	1.57	no	no	yes
	4	0x	36.3					
		10x	26.4	26.3	0.17	no	yes	no
		14x	22.5	22.3	0.29	no	yes	no
	5	0x	n/a					
		10x	30.5	n/a	n/a	no	no	yes
		14x	25.4	n/a	n/a	no	no	yes
	6	0x	n/a					
		10x	27.5	n/a	n/a	no	no	yes
		14x	23.6	n/a	n/a	no	no	yes
	7	0x	n/a					
		10x	27.9	n/a	n/a	no	no	yes
		14x	23.9	n/a	n/a	no	no	yes
	8	0x	36.1					
		10x	29.0	26.1	2.97	no	no	yes
		14x	24.1	22.1	1.98	no	no	yes
	9	0x	n/a					
		10x	n/a	n/a	n/a	no	no	yes
		14x	28.2	n/a	n/a	no	no	yes
	10	0x	n/a					
		10x	29.4	n/a	n/a	no	no	yes
		14x	25.8	n/a	n/a	no	no	yes
	11	0x	n/a					
		10x	n/a	n/a	n/a	no	no	yes
		14x	n/a	n/a	n/a	no	no	yes
	12	0x	n/a					
		10x	n/a	n/a	n/a	no	no	yes
		14x	n/a	n/a	n/a	no	no	yes

DDB2	mean	0x	36.1			no	no	yes
		10x	27.9	26.1	1.8			
		14x	24.5	22.1	2.33			

Genes	Sample ID	Pre-amplification	Measurements: Mean Ct values observed	Measurements: Mean Ct values expected	Difference observed vs expected	Interpretation: Manufacturer predicts success	Our findings: works?	Agreement?
FDXR	*1*	*0x*	n/a					
		10x	27.6	n/a	n/a			
		14x	23.0	23.6	-0.60	no	yes	no
	2	*0x*	36.7					
		10x	26.4	26.7	-0.26	no	yes	no
		14x	22.3	22.7	-0.35	no	yes	no
	3	*0x*	n/a					
		10x	28.7	n/a	n/a			
		14x	23.9	24.7	-0.72	no	yes	no
	4	*0x*	37.7					
		10x	27.2	27.7	-0.47	no	yes	no
		14x	22.7	23.7	-0.96	no	yes	no
	5	*0x*	n/a					
		10x	30.5	n/a	n/a			
		14x	26.1	26.5	-0.40	no	yes	no
	6	*0x*	37.6					
		10x	30.0	27.6	2.32	no	no	yes
		14x	24.4	23.6	0.73	no	yes	no
	7	*0x*	n/a					
		10x	28.6	n/a	n/a			
		14x	24.6	24.6	0.06	no	yes	no
	8	*0x*	n/a					
		10x	30.3	n/a	n/a			
		14x	25.9	26.3	-0.36	no	yes	no
	9	*0x*	n/a					
		10x	30.1	n/a	n/a			
		14x	25.5	26.1	-0.58	no	yes	no
	10	*0x*	n/a					
		10x	28.9	n/a	n/a			
		14x	25.2	24.9	0.31	no	yes	no
	11	*0x*	n/a					
		10x	n/a	n/a	n/a	no	no	yes
		14x	n/a	n/a	n/a	no	no	yes
	12	*0x*	n/a					
		10x	31.0	n/a	n/a	no	no	yes
		14x	n/a	27.0	n/a	no	no	yes

Genes	Sample ID	Pre-amplification	Mean Ct values observed	Mean Ct values expected	Difference observed vs expected	Manufacturer predicts success	Our findings: works?	Agreement?
FDXR	*mean*	*0x*	37.4			no	yes	no
		10x	28.4	27.4	1.02			
		14x	23.9	23.4	0.49			

Genes	Sample ID	Pre-amplification	Measurements			Interpretation		
			Mean Ct values		*Difference observed vs expected*	Manufacturer predicts success	Our findings: works?	Agreement?
			observed	*expected*				
GAPDH	***1***	***0x***	**31.4**					
		10x	**26.3**	21.4	4.92	yes	no	no
		14x	**23.4**	17.4	6.05	yes	no	no
	2	***0x***	**31.2**					
		10x	**25.7**	21.2	4.50	yes	no	no
		14x	**22.6**	17.2	5.34	yes	no	no
	3	***0x***	**31.9**					
		10x	**27.6**	21.9	5.72	yes	no	no
		14x	**24.7**	17.9	6.78	yes	no	no
	4	***0x***	**31.3**					
		10x	**27.0**	21.3	5.70	yes	no	no
		14x	**23.9**	17.3	6.55	yes	no	no
	5	***0x***	**35.4**					
		10x	**30.8**	25.4	5.41	no	no	yes
		14x	**27.5**	21.4	6.03	no	no	yes
	6	***0x***	**32.7**					
		10x	**28.4**	22.7	5.73	yes	no	no
		14x	**25.4**	18.7	6.69	yes	no	no
	7	***0x***	**38.1**					
		10x	**37.3**	28.1	9.20	no	no	yes
		14x	**34.5**	24.1	10.43	no	no	yes
	8	***0x***	**33.3**					
		10x	**28.6**	23.3	5.35	yes	no	no
		14x	**25.3**	19.3	5.98	yes	no	no
	9	***0x***	**35.3**					
		10x	**30.9**	25.3	5.53	no	no	yes
		14x	**27.7**	21.3	6.42	no	no	yes
	10	***0x***	**34.6**					
		10x	**30.3**	24.6	5.71	yes	no	no
		14x	**27.2**	20.6	6.62	yes	no	no
	11	***0x***	**n/a**					
		10x	**n/a**	n/a	n/a	no	no	yes
		14x	**n/a**	n/a	n/a	no	no	yes
	12	***0x***	**n/a**					
		10x	**n/a**	n/a	n/a	no	no	yes
		14x	**35.8**	n/a	n/a	no	no	yes

GAPDH	***mean***	***0x***	**33.5**			yes	no	no
		10x	**29.3**	23.5	5.78			
		14x	**26.2**	19.5	6.69			

Supplemental table 1

The table depicts qRT-PCR results (single raw threshold cycle [Ct]-values, duplicate measurements not shown) of four gene targets (*CDKN1A, FDXR, DDB2* and *GAPDH*) for all saliva samples. Shown are the measured/observed mean Ct values for unamplified samples as well as for samples after 10X and 14X pre-amplification. The column for expected mean Ct values shows the calculated Ct values when estimating a 100% linear and unbiased 10X and 14X pre-amplification (steps by 10 and 14, taking the mean Ct value without pre-amplification). The ΔCt $_{\text{(observed vs expected)}}$ is shown for each gene and each pre-amplification. We categorized the data by whether unbiased pre-amplification should have worked according to the manufacturer (Ct value without pre-amplification <35, yes) or not (Ct value without pre-amplification >35, no). For *FDXR* the interpretation whether we could show linear pre-amplification, when no Ct value was available without pre-amplification, was done according to the steps between 10X and 14X pre-amplification. The data are further presented if pre-amplification worked linear (yes) or not (no) according to the amplification biases through deviating expression patterns between amplified and un-amplified material.
